# Characteristics of Fecal Microbiota and Machine Learning Strategy for Fecal Invasive Biomarkers in Pediatric Inflammatory Bowel Disease

**DOI:** 10.3389/fcimb.2021.711884

**Published:** 2021-12-07

**Authors:** Xinqiong Wang, Yuan Xiao, Xu Xu, Li Guo, Yi Yu, Na Li, Chundi Xu

**Affiliations:** ^1^Department of Pediatrics, Ruijin Hospital, Shanghai Jiao Tong University, School of Medicine, Shanghai, China; ^2^Department of Molecular Medicine, University of Utah School of Medicine, Salt Lake City, UT, United States; ^3^Institute of Tropical Medicine, Hainan Medical University, HaiKou, China

**Keywords:** biomarkers, gut microbiome, inflammatory bowel disease, machine learning, pediatrics

## Abstract

**Background:**

Early diagnosis and treatment of pediatric Inflammatory bowel disease (PIBD) is challenging due to the complexity of the disease and lack of disease specific biomarkers. The novel machine learning (ML) technique may be a useful tool to provide a new route for the identification of early biomarkers for the diagnosis of PIBD.

**Methods:**

In total, 66 treatment naive PIBD patients and 27 healthy controls were enrolled as an exploration cohort. Fecal microbiome profiling using 16S rRNA gene sequencing was performed. The correlation between microbiota and inflammatory and nutritional markers was evaluated using Spearman’s correlation. A random forest model was used to set up an ML approach for the diagnosis of PIBD using 1902 markers. A validation cohort including 14 PIBD and 48 irritable bowel syndrome (IBS) was enrolled to further evaluate the sensitivity and accuracy of the model.

**Result:**

Compared with healthy subjects, PIBD patients showed a significantly lower diversity of the gut microbiome. The increased *Escherichia-Shigella* and *Enterococcus* were positively correlated with inflammatory markers and negatively correlated with nutrition markers, which indicated a more severe disease. A diagnostic ML model was successfully set up for differential diagnosis of PIBD integrating the top 11 OTUs. This diagnostic model showed outstanding performance at differentiating IBD from IBS in an independent validation cohort.

**Conclusion:**

The diagnosis penal based on the ML of the gut microbiome may be a favorable tool for the precise diagnosis and treatment of PIBD. A study of the relationship between disease status and the microbiome was an effective way to clarify the pathogenesis of PIBD.

## Introduction

The incidence of Inflammatory bowel disease (IBD) has grown rapidly worldwide (Molodecky et al., 2012; Wang et al., 2013). About 20-30% of patients develop IBD before adulthood (Oliveira and Monteiro, 2017). This is even more dramatic in China. In the past decade, the prevalence of Pediatric IBD (PIBD) in Shanghai has increased more than 10-fold. The Asian population has unique genetic and environmental signatures compared to Caucasian and other ethnic groups, including differences in their dietary habits and the constitution of the gut microbiome. The current guidelines for the management of PIBD are mainly based on evidence from studies involving Caucasian populations and a better understanding of the individual signature of Chinese PIBD patients is required for early precision diagnosis and individualized treatment in the future. Nevertheless, there have been few attempts in China to examine the systemic characterization of PIBD to date.

Gut microbiome has been proposed as a promising non-invasive diagnosis tool for PIBD in the last decade. The gut microbiota is the most important microecosystem in symbiosis with humans (Rooks and Garrett, 2016). PIBD patients often have dysregulated gut microbiota, including shifts in bacterial taxa constitution and diversity (Shaw et al., 2016). The microbiota diversity was decreased in patients with IBD *vs* controls according to a systematic review for both children and adults (Pittayanon et al., 2020). A meta study in PIBD showed that *Prevotella, Clostridium, Blautia*, and *Ruminococcus* were depleted while *Lactobacillus, Enterococcus*, and *Acidaminococcus* were increased in IBD (Knoll et al., 2017). Malham et al. found that *Akkermansia, Gemmiger, Ruminococcus*, and *Bacteroides* were decreased in PIBD (Malham et al., 2019). These studies mainly included European and North American populations. Whether this is consistent in the Chinese population is unclear. In the Chinese population, the microbiota signature in treatment naive PIBD patients and their role in the pathogenesis of PIBD remains largely to be addressed. Recently, Wang and colleagues reported the first gut microbiome profiling study of pediatric Crohn’s disease patients in a Chinese population (Wang et al., 2018b). They found different dysbiosis signatures in pediatric Crohn’s disease (CD) as compared to the reported Caucasian studies. According to their study, individual microbial signatures could be a useful tool for the prediction of a patient’s response to anti-TNF-α therapy. However, their study only included patients with a clear diagnosis of CD and anti-TNF-α treatment. Further studies including other types of IBD and the enrollment of treatment naive patients could provide valuable information for the early and differential diagnosis of PIBD.

Machine learning (ML), one of the most useful artificial intelligence (AI) for complex data statistics, has been successfully used for the diagnosis and early prediction of diseases such as cardiovascular diseases, cancer, and immune diseases (Rahman et al., 2018; Cammarota et al., 2020). It is worthwhile to explore its diagnostic value in PIBD.

In our study, 66 newly diagnosed young IBD patients and 27 healthy controls were prospectively enrolled for gut microbiome profiling. In addition, we also enrolled 48 patients with irritable bowel syndrome (IBS) for comparison to evaluate the performance of our diagnosis tool in the differential diagnosis. Fecal microbiota profiling was carried out using 16S ribosomal RNA gene sequencing (16S rRNAseq). The profiles of the microbiome in PIBD and its relationship with disease activity and nutrition status were analyzed. A diagnosis model for PIBD was constructed based on intestinal microecological machine learning. Our study presents comprehensive profiling of the gut microbiome and reveals unique biomarkers in Chinese PIBD patients. These insights into the complex interactions between the gut microbiome and hosts may also provide new insight into the pathogenesis of PIBD.

## Materials and Methods

### Study Cohort

In the initial discovery stage, we enrolled 66 IBD patients and 27 healthy controls for microbiome profiling. In the second validation stage, we enrolled 14 early-onset IBD and 48 IBS patients. All patients with IBD or IBS were recruited from the Department of Pediatrics, Ruijin Hospital affiliated with the School of Medicine, Shanghai Jiao Tong University from January 2016 to December 2019.

The diagnosis and disease evaluation were undertaking by following the protocol used in a previous study (Wang et al., 2018a). Briefly, for the diagnosis of CD, the Proto standard (Levine et al., 2014) was used and disease activity was assessed with the Pediatric Crohn’s Disease Activity Index (PCDAI) (Hyams et al., 2005); for patients with UC, the Pediatric Ulcerative Colitis Activity Index (PUCAI) was used (Turner et al., 2009); for the diagnosis of IBS, Rome IV Criteria was used (Stanghellini et al., 2016). The index score of height and weight was calculated using the Z-scoring method based on a national survey in China in 2005 (Li et al., 2009).

All patients enrolled were newly diagnosed children below 18 years old and without any treatment for IBD. Patients were excluded from the study if they met the following criteria: 1) the diagnosis changed and was not considered as IBD. 2) The patient had taken antibiotics in the month before collecting the fecal samples. 3) The patients and their guardians did not agree to take part in the study. The healthy control group had not taken antibiotics for at least one month before entry. Written informed consent was obtained from each participant following the protocols approved by the institutional review boards of the Shanghai Jiao Tong University.

### Sample Collection and DNA Extraction

Blood samples were collected in IBDs and commonly used clinical parameters were recorded, including white blood cells (WBC), platelets (PLT), C-reactive protein (CRP) and erythrocyte sedimentation rate (ESR), Neutrophil-to-Lymphocyte Ratio (NLR), Platelet-to-Lymphocyte ratio (PLR), albumin (ALB), hemoglobin (HGB), and hematocrit (HCT).

Fecal samples were collected from all participants and saved at −80°C within 3 hours. DNA extraction was performed using the E.Z.N.A.^®^ soil DNA Kit (Omega Bio-tek, Norcross, GA, U.S.A.) according to the manufacturer’s protocols. The final DNA concentration and purification were determined by NanoDrop 2000 UV-vis spectrophotometer (Thermo Scientific, Wilmington, DE, U.S.A.), and DNA quality was checked by 1% agarose gel electrophoresis. The concentrations of all samples were above 50ng/ul. 10ng of DNA was used for 16S rRNAseq. The OD value of 260/280 of all DNA samples was between 1.8~2.0 to confirm the quality of the samples.

### 16S rRNAseq

The V3-V4 hypervariable regions of the bacteria 16S rRNA gene were amplified with primers 338F (5’-ACTCCTACGGGAGGCAGCAG-3’) and 806R (5’-GGACTACHVGGGTWTCTAAT-3’) by thermocycler PCR system GeneAmp 9700 (Thermo Scientific). The PCR products were purified using the AxyPrep DNA Gel Extraction Kit (Axygen Biosciences, Union City, CA, USA) and quantified using QuantiFluor™-ST (Promega, Madison, WI, U.S.A.) according to the manufacturer’s protocol. Purified amplicons were pooled in equimolar and paired-end sequenced (2×300 cycle run) on an Illumina MiSeq platform (Illumina, San Diego, CA, U.S.A.) according to the standard protocols outlined by Majorbio Bio-Pharm Technology Co. Ltd. (Shanghai, China).

### Processing of Sequencing Data

The raw fastq files were demultiplexed, quality filtered by Trimmomatic, and merged by FLASH. Operational taxonomic units (OTUs) were clustered with 97% similarity cutoff using UPARSE (version 7.1 http://drive5.com/uparse/) and chimeric sequences were identified and removed using UCHIME. The taxonomy of each 16S rRNA gene sequence was analyzed by the Silva128/16s bacteria database using a confidence threshold of 70%.

The 16S rRNA data were further analyzed and visualized on the online Majorbio Cloud Platform (www.majorbio.com). Alpha-diversity analyses, including community richness parameters (Sobs, Chao) and community diversity parameters (Shannon). Beta diversity measurements, including principal coordinate analyses (PCoA) and Partial Least Squares Discriminant Analysis (PLS-DA) based on OTU compositions, were determined. The bacterial taxonomic distributions of sample communities were visualized. Linear discriminant analysis effect size (LEfSe) was conducted to identify OTUs differentially.

### OTU-Based Biomarkers Identification

Random forest models (Random Forest 4.6-14 package) were used to model OTU-based biomarkers as described before (Edwards et al., 2018). Briefly, we ranked individual OTUs by their importance. 10-fold cross validation was performed to evaluate model performance as well as to remove less important OTUs. The top 11 OTUs from the random forest models were listed with the smallest number of OTUs as the optimal set. The probability of disease (POD) for IBD in both the exploration and validation cohort were calculated and compared. To evaluate the discriminatory ability of the random forest models, operating characteristic curves (receiving operational curve, ROC) were constructed and the area under the curve (AUC) was calculated.

### Imputed Metagenomic Analysis

The metagenomes of gut microbiota were imputed from 16S rRNAseq with Tax4Fun package available on Majorbio Cloud Platform. The predicted functional composition profiles were collapsed into KEGG (Kyoto Encyclopedia of Genes and Genomes) database pathways.

### Statistical Analysis

The free online platform of Majorbio Cloud Platform (www.majorbio.com) or GraphPad 8.0 (GraphPad Software Inc, CA) was used for statistics. For the comparison of continuous variables, the Mann-Whitney U test for two groups was used. For correlation analysis, Spearman’s rank test was performed. Multiple hypothesis tests were adjusted using Benjamini and Hochberg false discovery rate (FDR), and significant association was considered below an FDR threshold of 0.05. The differences between populations were analyzed using a one-way ANOVA. P < 0.05 was considered statistically significant.

## Results

### Characteristics of the Participants

We recruited a total of 66 subjects with IBD and 27 healthy control subjects as the exploration group. Another 14 IBD and 48 IBS patients were enrolled for the evaluation of the diagnosis model. All the patients were newly diagnosed with PIBD. The demographic and clinical characteristics of PIBD and non-IBD controls are shown in **Table 1**.

**Table 1:** Demographic characteristics of study subjects at diagnosis.

	Exploration		Validation	
	IBD (n = 66)	Healthy control (n = 27)	IBD (n = 14)	IBS (n = 48)
Age, Mean ± SD, years	10.0 ± 5.3	7.1 ± 3.8	2.3 ± 3.1	6.7 ± 3.5
Gender, male (%)	40 (60.6)	13 (48.1)	8 (57.1)	22 (45.8)
HAZ	-0.76 ± 1.46	0.13 ± 0.80		
WAZ	-0.67 ± 1.09	0.36 ± 1.16		
Disease type, n(%)				
CD	54 (81.8)		12 (85.7)	
UC	8 (12.1)		2 (14.3)	
IBDU	4 (6.1)			

HAZ, z score for height of age; WAZ, z score for weight of age; IBD, inflammation bowel disease; CD, Crohn’s disease; UC, ulcerative colitis; IBS, irritable bowel syndrome.

### Gut Microbial Dysbiosis in PIBD

**Figure 1:**
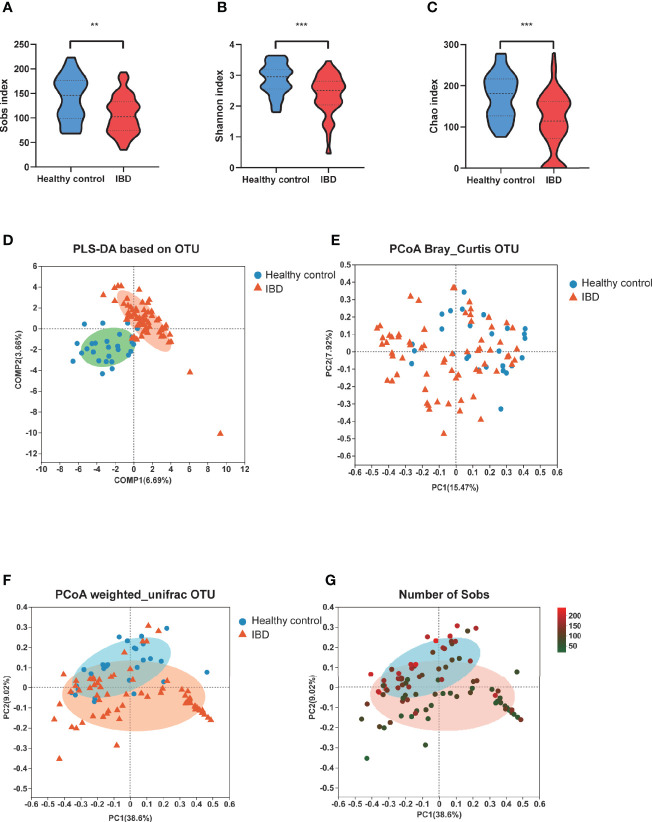
Changes of fecal microbial diversities: IBD (n=66) compared with healthy controls (n=27). α-Diversity illustrated by microbiota richness number of observed operational taxonomic unit (OTU) and evenness Sobs index **(A)**, Shannon index **(B)** and Chao **(C)** were reduced in IBD (**p<0.01, ***p<0.001). **(D)** showed the PLS-DA based on OTU compositions. **(E)** PCoA demonstrated under Bray Curtis distance (F model: 3.78, P<0.001) **(F)** PCoA demonstrated under weighted unifric that individuals with IBD were different from healthy controls. **(G)** The same PCoA plot as F colored by α-diversity measured by the number of Sobs index.

To investigate the gut microbiome in our PIBD cohort, fecal samples from all 66 PIBD patients and 27 healthy controls were processed for 16S rRNAseq. Consistent with the findings reported in Caucasian populations, the gut microbiota α-diversity was significantly reduced in our Chinese PIBD cohort, including decreases in the Sobs index, Shannon index, and Chao index (**Figures 1A–C**). This suggests not only a significantly decreased number of bacterial species but also less evenly distributed species in PIBD. Beta diversity was also calculated to compare the similarity of bacteria species between the two groups. PERMANOVA analysis under Bray Curtis distance was further calculated. (F model: 3.78, P<0.001) (**Figures 1D–F**). This suggested asymmetrical distribution between the two groups. Notably, we found that the richness of species could explain the differences along the principal coordinate by Weighted Unifrac PCoA analysis (**Figure 1G**).

**Figure 2:**
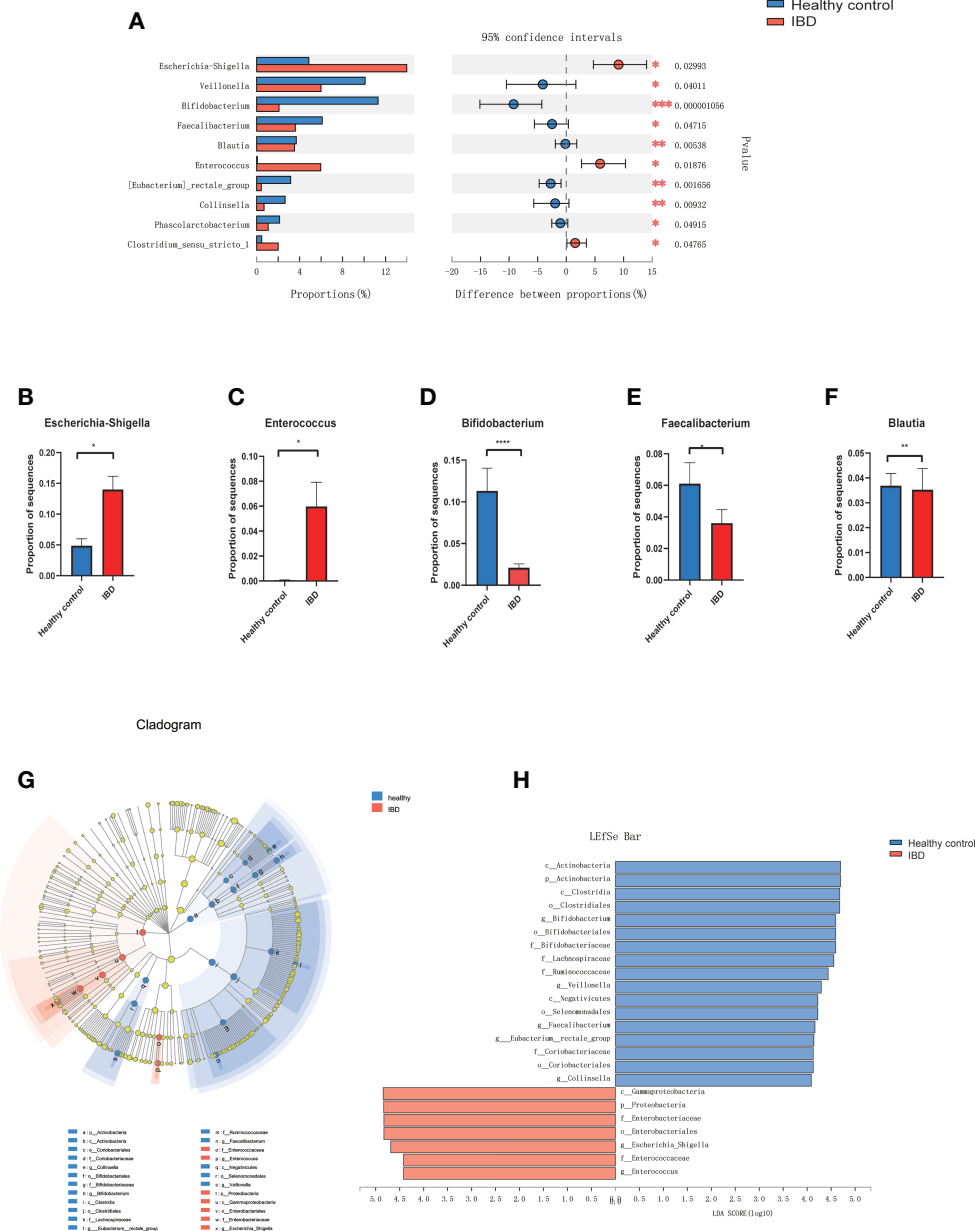
Microbiome alterations at the genus level in IBD. **(A)** The relative abundance of 10 genera were significantly different between IBD (n=66) and healthy controls (n=27) after correcting for confounding variables. **(B–F)** showed the difference of *Bifidobacterium, Escherichia-Shigella, Enterococcus, Faecalibacterium*, and *Blautia* between IBD and healthy control. **(G)** Cladogram generated by Lefse analysis showing enriched taxa between the IBD and healthy controls; **(H)** LDA scores of enriched taxa from G (LDA>4). (*p<0.05, **p<0.01, ***p<0.001, ****p<0.0001). Linear discriminant analysis (LDA); LDA effect size algorithm (LEfSe).

At the phylum level, Proteobacteria was significantly increased in PIBD patients (P=0.0014) while Actinobacteria was decreased in PIBD patients as compared with healthy controls (P<0.0001). **Figure 2A** shows the most significantly altered 10 genera between PIBD and healthy controls. *Escherichia-Shigella* and *Enterococcus* were enriched in PIBD patients (**Figures 2B, C**). *Bifidobacterium, Faecalibacterium*, and *Blautia* were decreased in PIBD patients (**Figures 2D–F**). The linear discriminant analysis effect size algorithm (LEfSe) analysis results in **Figures 2G, H** further show significantly different signatures between the two groups (**Figures 2G, H**).

### The Association Between Microbiome With the Disease Status

**Figure 3:**
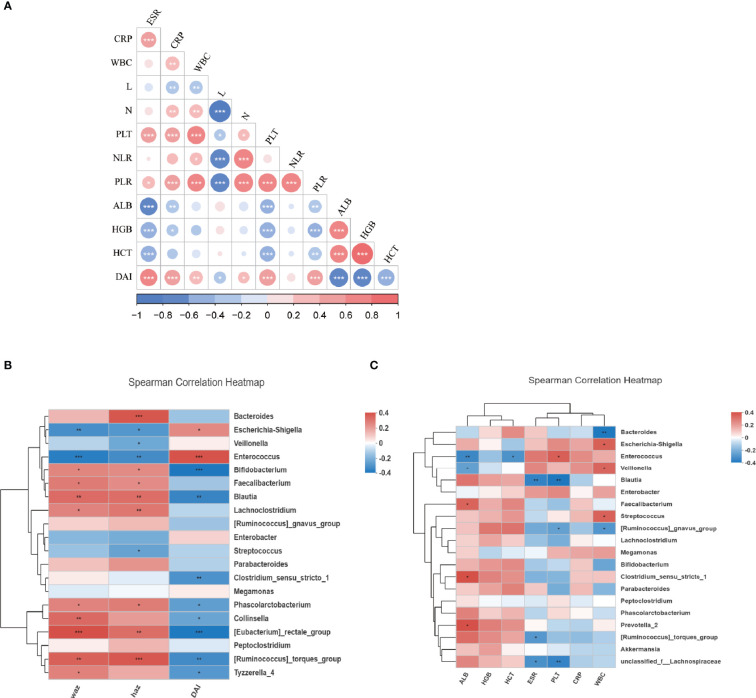
The relationship of microbiota and clinical characteristics in IBDs. **(A)** showed the Spearman analysis of inflammatory markers, nutrition markers, and the DAI score. A positive correlation is shown in red and a negative correlation is shown in blue. **(B)** A heatmap of Spearman correlation between microbiota at genus level and DAI, WAZ, and HAZ. **(C)** A heatmap of Spearman correlation between microbiota at genus level and inflammatory markers (WBC, CRP, PLT, ESR, NLR, PLR) and nutrition markers (ALB, HGB, HCT). IBD, inflammation bowel disease; DAI, disease activity index; HAZ, z score for height of age; WAZ, z score for weight of age; WBC, white blood cells; PLT, platelets; CRP, C-reactive protein; ESR, erythrocyte sedimentation rate; N, neutrophil; L, lymphocyte; NLR, neutrophil-to-lymphocyte ratio; PLR, platelet-to-lymphocyte ratio; ALB, albumin; HGB, hemoglobin; HCT, hematocrit. *P < 0.05, **P < 0.01, ***P < 0.001.

We next evaluated the relationship between the composition of genera with the disease severity in IBDs. For disease activity assessment, the PCDAI score was used for patients with CD. The mean index of PCDAI is 14.5 ± 17.1. For patients with Ulcerative Colitis (UC), the PUCAI score was used and the mean index is 16.9 ± 12.5. We combined the two indexes as Disease Activity Index (DAI) in the further statistics. The detail of the patient’s disease severity and nutrition status are listed in **Table 2**. Spearman analysis showed DAI was positively correlated with several inflammatory markers including ESR, CRP, WBC, PLT, PLR. DAI also negatively correlated with the nutrition markers, including ALB, HGB, and HCT (**Figure 3A**). In addition, several inflammatory markers are positively or negatively associated with each other. For example, PLR is significantly associated with every other parameter, including positive correlation with ESR, CRP, WBC, Neutrophil, PLT NLR, DAI; and negative correlation with lymphocyte count, ALB, HGB, and HCT.

**Table 2:** Disease severity and nutrition status of PIBD patients.

Disease sore	Mean ± SD, n=66	Normal Range
PCDAI (n=58)	14.5 ± 17.1	
PUCAI (n=8)	16.9 ± 12.5	
Inflammatory markers	Mean ± SD, n=62	
WBC (10^9^/L)	8.2 ± 4.7	3.97 - 9.15
PLT (10^9^/L)	343.3 ± 137.7	85 - 303
CRP (mg/L)	15.8 ± 29.7	<10
ESR (mm/h)	15.1 ± 11.5	<20
NLR	2.0 ± 1.7	1.75 - 2.50
PLR	11.8 ± 8.4	4.25 - 7.57
ANCA+	2/47(4.3%)	Negative
Nutrition markers	Mean ± SD, n=62	
HGB (g/L)	116.9 ± 15.8	120 - 172
HCT	46.7 ± 112.0	38.0 - 50.8
ALB (g/L)	36.2 ± 5.0	35 - 52

PIBD, pediatric inflammation bowel disease; PCDAI, pediatric Crohn’s disease activity index; PUCAI, pediatric ulcerative colitis activity index; WBC, white blood cells; PLT, platelets; CRP, C-reactive protein; ESR, erythrocyte sedimentation rate; NLR, neutrophil-to-lymphocyte ratio; PLR, platelet-to-lymphocyte ratio; ALB, albumin; HGB, hemoglobin; HCT, hematocrit; ANCA, Anti-neutrophil cytoplasmic antibodies.

The correlation between the disease activity and microbiome profile was further examined using DAI score and 16S rRNAseq data. As revealed by Spearman analysis, the richness of *Enterococcus, Escherichia-Shigella, Streptococcus, Enterobacter*, and *Veillonella* were positively associated with higher DAI score and inflammatory markers, and negatively correlation nutrition markers. Moreover, dysbiosis in IBD was also negatively associated with the Z score of height and weight, suggesting the host gut dysbiosis leads to an exaggerated disease. Oppositely, *Faecalibacterium, Lachnoclostridium, Bacteroides, Parabacteroides, Blautia*, and *Prevotella* were positively correlated with the Z score of height and/or weight and nutrition markers but negatively correlated with DAI and inflammatory markers. This suggests that microbiota were associated with favorable outcomes in patients (**Figures 3B, C**).

### Differential Diagnosis Discrimination With 11 OTUs Signature

**Figure 4:**
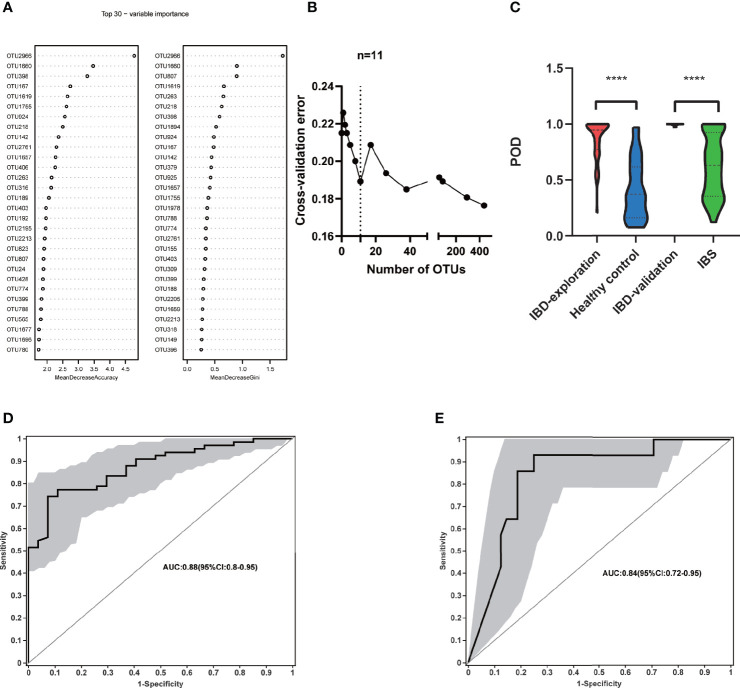
Identification of microbial based markers of IBD by random forest models. **(A)** showed the top 30 main important IBD-associated OTU from random forest models. **(B)** The top 11 OTUs markers were selected as the optimal marker set by random forest models. **(C)** The POD value was significantly increased in the IBD samples *versus* the healthy control and IBS. Receiving operational curve analysis was performed in **(D)** exploration data (IBD: 66; control: 27, area under curve (AUC)=0.88) and in **(E)** validation data (IBD:14; IBS:48, AUC=0.84), respectively. Diagonal lines represent random classification (AUC=0.5). ****P<0.0001, AUC, area under the curve; IBD, inflammatory bowel disease; IBS, irritable bowel syndrome; OTUs, operational taxonomy units; POD, probability of disease.

To explore the diagnostic value of fecal microbiome profiling in PIBD, we applied the ML approach to analyze the major factors for the diagnosis of PIBD. We constructed a random forest model based on the total 1902 OTUs of gut microbiota in the exploration group. The top 30 OTUs were then ranked using the index of accuracy and Gini (**Figure 4A**). 11 OTUs were further collected by 10-fold cross validation as the optimal marker set (**Figure 4B**). These OTUs were mainly from the genus of *Bifidobacterium* (OTU2966, OTU218)*, Ruminiclostridium* (OTU1660)*, Sphingobium* (OTU398), *Anaerostipes* (OTU167), *Fusicatenibacter* (OTU1619), *Clostridium* (OTU1755), *Brevundimonas* (OTU924)*, Lachnospriaceae* (OTU142)*, Adlercreutzia* (OTU2761), and *Dorea* (OTU1657). The POD index was generated using Random Forest model analysis. It showed significantly increased value in PIBD samples versus healthy control (P<0.0001) (**Figure 4C**).

The POD index was further examined using microbial data from the validation cohort. In addition to the comparison between healthy controls and PIBD patients to generate an unbiased POD index, it is even more important to identify PIBD at an early stage from other diseases with similar symptoms. To better evaluate its effectiveness in the diagnosis and differential diagnosis of IBD, we included patients with IBS rather than healthy controls in the validation cohort. This is because clinically it is more relevant to differentiate IBD from other patients with similar symptoms at an early stage than differentiating the IBD from healthy controls. The POD index was significantly higher in IBD samples than IBS ones (P<0.0001) (**Figure 4C**). The performance of the model was assessed using ROC analysis, the exploration group achieved an AUC value of 0.88 (95% CI:0.8-0.95) (**Figure 4D**). AUC was 0.84 in the validation cohort (95% CI:0.72-0.96) (**Figure 4E**). This result indicated that the gut microbiome-based classifier can accurately and sensitively distinguish IBD from Non-IBD. Therefore, fecal invasive biomarkers obtained by ML achieved a powerful diagnostic potential for IBD.

### Microbial Functions Altered in PIBD

**Figure 5:**
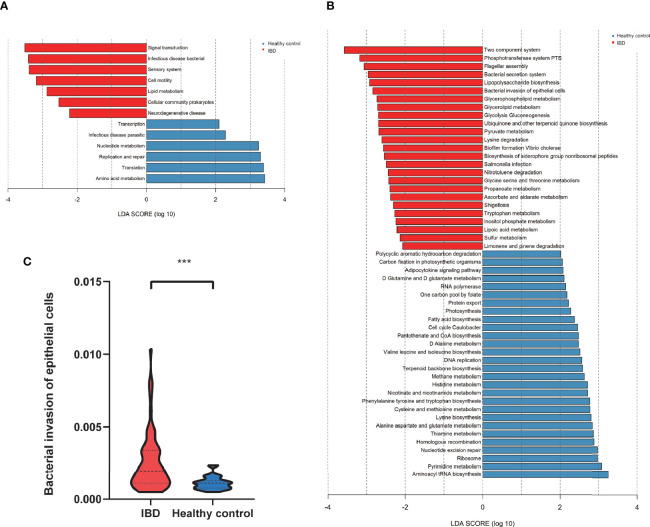
The predicted functional module, based on gut microbiota. **(A)** KEGG pathway level2 analysis based on 16S rRNA (LDA>3). **(B)** KEGG pathway level 3 analysis based on 16S rRNA (LDA>2). **(C)** The abundant bacterial invasion of epithelial cells pathway between IBD and healthy control. ***P<0.001.

The functional profiles of the gut microbiome in IBD patients and healthy controls were predicted with Tax4Fun based on the 16S rRNAseq data. The KEGG pathway analysis predicted that bacterial invasion of the epithelial cells pathway was significantly altered in IBD (**Figures 5A–C**). The gut microbiome of IBD was characterized by over-representation of pathogenetic bacteria. In contrast, the pathway enriched in healthy controls highlighted pathways in “replication and repair”, “amino acid metabolism” and “nucleotide metabolism” in level 2 KEGG pathway analysis (**Figure 5A**). Differentially expressed level 3 KEGG pathways are listed in **Figure 5B**. The “abundant bacterial invasion of epithelial cells” pathway was significantly increased in IBD patients (**Figure 5C**).

**Figure 6:**
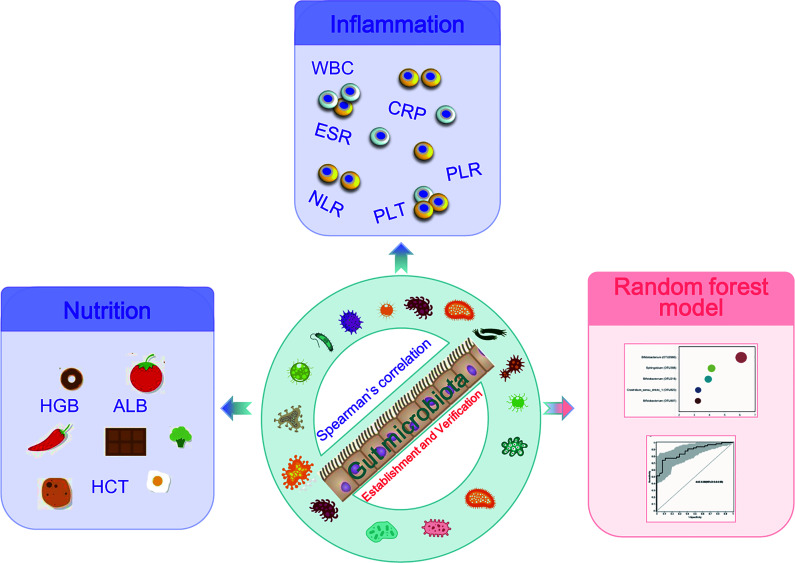
Study design: discovery cohort enrolled 66 newly diagnosed young IBD patients and 27 healthy controls for gut microbiome 16S rRNAseq. The profiles of the microbiome in PIBD and its relationship with the disease activity and nutrition status were analyzed. Intestinal microecological machine learning was constructed to generate a PIBD diagnosis tool using microbiome data. Subsequently, 14 patients with IBD and 48 patients with IBS were collected as a validation cohort for the evaluation of the diagnostic model.

## Discussion

We studied the microbiota of Chinese PIBD patients using 16S rRNAseq. PIBD patients showed significantly lower diversity of their gut microbiome as compared to healthy controls. They had increased Escherichia-Shigella and Enterococcus, which were positively correlated with inflammatory markers, but were negatively correlated with nutrition markers. Bifidobacterium, Faecalibacterium, and Blautia were decreased in IBD patients. A diagnostic model was successfully developed integrating 11 OTUs using ML methods for differential diagnosis in PIBD. This diagnostic model showed outstanding performance differentiating IBD from IBS in an independent validation cohort.

PIBD is a chronic gastrointestinal disease. The main challenge in the diagnosis of PIBD is the occult onset of the disease. Non-invasive early diagnostic tools would provide possibilities for early intervention and improve the quality of life of the patients. Several studies have shown that, through ML-based predictive models, the microbial markers outperform clinical parameters in the diagnosis and prediction of relapse and response to therapies (Ananthakrishnan et al., 2017; Zhou et al., 2018; Aden et al., 2019). However, the gut microbiome significantly differs between the Chinese and Caucasian populations (Zhou et al., 2018). Moreover, Chinese PIBD patients also have different disease progress and different underlying pathogenesis, such as IL10-RA gene polymorphism, as we and others have shown (Wang et al., 2018a; Su et al., 2021). Therefore, the previously reported tools could not be directly applied to our PIBD patient cohort. We believe the exploration of microbiota and ML-based diagnostic tools will help the early diagnosis and therapy for PIBD in China. Our current study provides the first successful diagnostic model of microbial OTUs markers for PIBD in the Chinese population.

In our study, *Escherichia-Shigella* and *Enterococcus* were enriched in IBD patients *Escherichia-Shigella* is the specific *Escherichia coli* strain. Pathogenic *E. coli* can evade the immune system of the host and induce inflammation by suppressing epithelial and inflammatory cell autophagy (Schirmer et al., 2019). Recurrent infection of *Salmonella* can cause colitis by accelerating molecular aging (Yang et al., 2017). *Enterococcus* can also trigger pathological processes in IBD (Mancabelli et al., 2017; Wang et al., 2018b; Lo Presti et al., 2019; Salem et al., 2019). Zhou et al. found that *Enterococcus faecalis* (*E. faecalis*) levels were associated with clinically active disease in patients with CD (Zhou et al., 2016). The gelatinase from *E. faecalis*. can disrupt the intestinal epithelium by activating the protease-activated receptor 2 (Maharshak et al., 2015).

A reduction of Short Chain Fatty Acid (SCFA) producing microbiota including *Faecalibacterium, Blautia, Clostridium*, and *Lachnospiraceae* in PIBD samples was found in the study. SCFA has been regarded as a source of energy for epithelium and can protect the tight junction of epithelial cells (Fachi et al., 2019). SCFA can also be attributed to active regulatory T cell function (Arpaia et al., 2013) and reduce neutrophils recruitment through blockade of IL-8 production (Sokol et al., 2008).

The high richness of *Escherichia-Shigella* and *Enterococcus* in PIBD were positively associated with disease severity. In contrast, the microbiota lost in PIBD, such as *Bifidobacterium, Faecalibacterium*, and *Blautia* were related to the good nutrition status and low disease score. Xue et al. also reported a microbial dysbiosis in pediatric CD, with increased *Entrococcus, Novosphingobium*, and *Enhydrobacter* and decreased *Bifidobacterium, Klebsiella*, and *Closridium* (Xue et al., 2020). Wang et al. had consistent findings (Wang et al., 2021). PIBD in China is characterized by increased *Entrococcus* and decreased *Bifidobacterium Faecalibacterium* and *Blautia*, which indicates that the microbiome of PIBD patients within the Chinese population have some conserved features. This might be associated with genetic or environmental factors and provides the foundation for microbiome-based diagnosis and disease evaluation.

Disease severity is significantly associated with inflammatory markers in the peripheral blood and nutritional status in children. It is generally accepted that elevated inflammation markers often indicate active disease, and the restoration of nutritional markers indicates that the patient has a more stable status (van Rheenen et al., 2021). This is also consistent with our current study. In this study, the differential microbiota was closely related to the disease severity, which indicates that the changing of the microbiome can be biomarkers that reflect disease severity and predict the outcome before clinical syndromes. Sylvie et al. reported reduced counts of *Clostridium* and *Faecalibacterium* in CD patients and a lower baseline abundance of *F. prausnitzii* and *Bacteroides* predicted relapse (Rajca et al., 2014). Hyams et al. found that the abundance of Ruminococcaceae and Sutterella predicted remission in 400 newly diagnosed Pediatric UC (Hyams et al., 2019). In a PIBD study, the enrichment of *Rothia* and *Ruminococcus* was associated with the development of strictured complications (Kugathasan et al., 2017). The microbiome-based biomarkers could well predict the disease progression or outcome.

AI has revolutionized the study of IBD (Iablokov et al., 2020). According to the Random Forest models, the optimal 11 OTUs markers for PIBD were identified. The POD based on the 11 OTUs markers were distinct between PIBD and healthy controls, which achieved powerful classification potential for PIBD. More importantly, the POD successfully achieved validation of patients with IBD from IBS. Similarly, the study in adult patients also successfully demonstrated ML modeling for IBD using gut microbiome data in the United States (Manandhar et al., 2021). Xu et al. constructed a gut microbiome-based diagnostic tool for differential diagnosis and achieved a high AUC value both in health *vs* IBD and UC *vs* CD (Xu et al., 2021). These findings indicated that ML approaches offer the ability to improve the accuracy and convenience of diagnosing IBD, and fecal microbial markers show promising potentials as non-invasive tools for the early diagnosis of PIBD. However, our initial study with ML-based tools also has limitations that require further investigation. For example, we designed this tool for the diagnosis of typical PIBD at an early stage, and therefore we could not include patients who mainly have extraintestinal manifestations. Whether this tool could be improved to identify non-typical PIBD remains to be validated. Although machine learning methods are still evolving, it shows promising power in both early diagnosis and identifying underlying pathogenesis that can guide mechanistic studies. Nonetheless, Microbiome-based biomarkers have an irreplaceable advantage and continued exploration is needed to accelerate the applications of precision medicine.

Using the functional prediction of 16S sequencing, we found IBD patients have enriched strains of pathological bacteria and decreased diversity of the microbiota related to the host’s metabolic capacities. Pathway analysis revealed significantly upregulated pathways in the bacterial invasion of epithelial cells in our PIBD patients. Not surprisingly, this was closely related to the altered host microbiome in PIBD patients, featuring changes in *Escherichia-Shigella, Enterococcus*, and SCFA-producing microbiota (Solis et al., 2020). Another potential contributing factor is the host diet, as diet was recently found able to influence the human gut microbiome and the pathogenesis of IBD (Adithya et al., 2021). A deeper investigation of the key species by metagenomic and metabolic sequencing may further improve our understanding of the early triggers in PIBD, enhance the performance of the diagnosis model, and provide new routes for the treatment of IBD.

## Conclusion

This study found dysbiosis in gut microbiota in PIBD. *Escherichia-Shigella* and *Enterococcus* were positively associated with the disease severity of PIBD. In contrast, *Bifidobacterium, Faecalibacterium*, and *Blautia* were associated with low inflammatory markers and good nutrition status. AI analysis of gut microbiota using ML models successfully identified optimal 11 OTUs biomarkers (OTU2966, OTU218, OTU1660, OTU398, OTU167, OTU1619, OTU1755, OTU924, OTU142, OTU2761, and OTU1657) for the diagnosis of PIBD, which could be potentially non-invasive tools for the early diagnosis of PIBD (**Figure 6**).

## Data Availability Statement

The datasets presented in the study are deposited in the Genome Sequence Archive (Chen et al., 2021) in National Genomics Data Center (Members and Partners, 2021), China National Center for Bioinformation/Beijing Institute of Genomics, Chinese Academy of Sciences (GSA: CRA005251) that are publicly accessible at https://ngdc.cncb.ac.cn/gsa.

## Ethics Statement

The studies involving human participants were reviewed and approved by Ethics committee of Ruijin Hospital, Shanghai Jiao Tong University School of Medicine. Written informed consent to participate in this study was provided by the participants’ legal guardian.

## Author Contributions

XW and YX contributed equally to this work. XW, YX, and CX designed this study. XW and NL analyzed the data. YY and XX collected the patients and recorded the data. XW, NL, and LG drafted the manuscript. CX and NL provided final approval of the manuscript. All authors contributed to the article and approved the submitted version.

## Funding

This work was supported by the National Natural Science Foundation of China, grant No. 81400588 to XW; Shanghai Science and Technology Funds, grant No. 19411971300 to CX; and the Jin Lei Pediatric Endocrinology Growth Research Fund for Young Physicians, grant No. PEGRF201809004 to YX.

## Conflict of Interest

The authors declare that the research was conducted in the absence of any commercial or financial relationships that could be construed as a potential conflict of interest.

## Publisher’s Note

All claims expressed in this article are solely those of the authors and do not necessarily represent those of their affiliated organizations, or those of the publisher, the editors and the reviewers. Any product that may be evaluated in this article, or claim that may be made by its manufacturer, is not guaranteed or endorsed by the publisher.

## Abbreviations

16S rRNAseq, 16S ribosomal RNA, gene sequencing; ALB, albumin; ANCA, Anti-neutrophil cytoplasmic antibodies; AUC, area under curve; CD, Crohn’s disease; CRP, C-reactive protein; DAI, Disease Activity Index; ESR, erythrocyte sedimentation rate; FDR, false discovery rate; HAZ, z score for height of age; HCT, hematocrit; HGB, hemoglobin; IBD, Inflammatory Bowel Disease; IBS, Irritable Bowel Syndrome; LEfSe, Linear discriminant analysis effect size algorithm; KEGG, Kyoto Encyclopedia of Genes and Genomes; ML, machine learning; NLR, Neutrophil-to-Lymphocyte Ratio; OTU, Operational taxonomic unit; PCDAI, Pediatric Crohn’s Disease Activity Index; PIBD, pediatric Inflammatory Bowel Disease; PLR, Platelet-to-Lymphocyte ratio; PLT, platelets; POD, probability of disease; PUCAI, Pediatric Ulcerative Colitis Activity Index; ROC, receiving operational curve; SCFA, Short Chain Fatty Acid; UC, Ulcerative Colitis; WAZ, z score for weight of age; WBC, white blood cells.

## References

[B1] Aden K. Rehman A. Waschina S. Pan W. H. Walker A. Lucio M.. (2019). Metabolic Functions of Gut Microbes Associate With Efficacy of Tumor Necrosis Factor Antagonists in Patients With Inflammatory Bowel Diseases. Gastroenterology 157 (5), 1279–1292.e1211. doi: 10.1053/j.gastro.2019.07.025 31326413

[B2] Adithya K. K. Rajeev R. Selvin J. Seghal Kiran G. (2021). Dietary Influence on the Dynamics of the Human Gut Microbiome: Prospective Implications in Interventional Therapies. ACS Food Sci. Technol. 1 (5), 717–736. doi: 10.1021/acsfoodscitech.0c00075

[B3] Ananthakrishnan A. N. Luo C. Yajnik V. Khalili H. Garber J. J. Stevens B. W.. (2017). Gut Microbiome Function Predicts Response to Anti-Integrin Biologic Therapy in Inflammatory Bowel Diseases. Cell Host Microbe 21 (5), 603–610.e603. doi: 10.1016/j.chom.2017.04.010 28494241 PMC5705050

[B4] Arpaia N. Campbell C. Fan X. Dikiy S. van der Veeken J. deRoos P.. (2013). Metabolites Produced by Commensal Bacteria Promote Peripheral Regulatory T-Cell Generation. Nature 504 (7480), 451–455. doi: 10.1038/nature12726 24226773 PMC3869884

[B5] Cammarota G. Ianiro G. Ahern A. Carbone C. Temko A. Claesson M. J.. (2020). Gut Microbiome, Big Data and Machine Learning to Promote Precision Medicine for Cancer. Nat. Rev. Gastroenterol. Hepatol. 17 (10), 635–648. doi: 10.1038/s41575-020-0327-3 32647386

[B6] Chen T. Chen X. Zhang S. Zhu J. Tang B. Wang A.. (2021). The Genome Sequence Archive Family: Toward Explosive Data Growth and Diverse Data Types. Genomics Proteomics Bioinformatics 13, 2021.08.001. doi: 10.1016/j.gpb.2021.08.001 PMC9039563 34400360

[B7] Edwards J. A. Santos-Medellin C. M. Liechty Z. S. Nguyen B. Lurie E. Eason S.. (2018). Compositional Shifts in Root-Associated Bacterial and Archaeal Microbiota Track the Plant Life Cycle in Field-Grown Rice. PloS Biol. 16 (2), e2003862. doi: 10.1371/journal.pbio.2003862 29474469 PMC5841827

[B8] Fachi J. L. Felipe J. S. Pral L. P. da Silva B. K. Corrêa R. O. de Andrade M. C. P.. (2019). Butyrate Protects Mice From Clostridium Difficile-Induced Colitis Through an HIF-1-Dependent Mechanism. Cell Rep. 27 (3), 750–761.e757. doi: 10.1016/j.celrep.2019.03.054 30995474

[B9] Hyams J. S. Davis Thomas S. Gotman N. Haberman Y. Karns R. Schirmer M.. (2019). Clinical and Biological Predictors of Response to Standardised Paediatric Colitis Therapy (PROTECT): A Multicentre Inception Cohort Study. Lancet 393 (10182), 1708–1720. doi: 10.1016/s0140-6736(18)32592-3 30935734 PMC6501846

[B10] Hyams J. Markowitz J. Otley A. Rosh J. Mack D. Bousvaros A.. (2005). Evaluation of the Pediatric Crohn Disease Activity Index: A Prospective Multicenter Experience. J. Pediatr. Gastroenterol. Nutr. 41 (4), 416–421. doi: 10.1097/01.mpg.0000183350.46795.42 16205508

[B11] Iablokov S. N. Klimenko N. S. Efimova D. A. Shashkova T. Novichkov P. S. Rodionov D. A.. (2020). Metabolic Phenotypes as Potential Biomarkers for Linking Gut Microbiome With Inflammatory Bowel Diseases. Front. Mol. Biosci. 7, 603740. doi: 10.3389/fmolb.2020.603740 33537340 PMC7848230

[B12] Knoll R. L. Forslund K. Kultima J. R. Meyer C. U. Kullmer U. Sunagawa S.. (2017). Gut Microbiota Differs Between Children With Inflammatory Bowel Disease and Healthy Siblings in Taxonomic and Functional Composition: A Metagenomic Analysis. Am. J. Physiol. Gastrointest Liver Physiol. 312 (4), G327–g339. doi: 10.1152/ajpgi.00293.2016 28039159

[B13] Kugathasan S. Denson L. A. Walters T. D. Kim M. O. Marigorta U. M. Schirmer M.. (2017). Prediction of Complicated Disease Course for Children Newly Diagnosed With Crohn's Disease: A Multicentre Inception Cohort Study. Lancet 389 (10080), 1710–1718. doi: 10.1016/s0140-6736(17)30317-3 28259484 PMC5719489

[B14] Levine A. Koletzko S. Turner D. Escher J. C. Cucchiara S. de Ridder L.. (2014). ESPGHAN Revised Porto Criteria for the Diagnosis of Inflammatory Bowel Disease in Children and Adolescents. J. Pediatr. Gastroenterol. Nutr. 58 (6), 795–806. doi: 10.1097/MPG.0000000000000239 24231644

[B15] Li H. Ji C. Y. Zong X. N. Zhang Y. Q. (2009). Height and Weight Standardized Growth Charts for Chinese Children and Adolescents Aged 0 to 18 Years. Zhonghua Er Ke Za Zhi 47 (7), 487–492. doi: 10.3760/cma.j.issn.0578-1310.2009.07.003 19951507

[B16] Lo Presti A. Zorzi F. Del Chierico F. Altomare A. Cocca S. Avola A.. (2019). Fecal and Mucosal Microbiota Profiling in Irritable Bowel Syndrome and Inflammatory Bowel Disease. Front. Microbiol. 10, 1655. doi: 10.3389/fmicb.2019.01655 31379797 PMC6650632

[B17] Maharshak N. Huh E. Y. Paiboonrungruang C. Shanahan M. Thurlow L. Herzog J.. (2015). Enterococcus Faecalis Gelatinase Mediates Intestinal Permeability *via* Protease-Activated Receptor 2. Infect. Immun. 83 (7), 2762–2770. doi: 10.1128/IAI.00425-15 25916983 PMC4468563

[B18] Malham M. Lilje B. Houen G. Winther K. Andersen P. S. Jakobsen C. (2019). The Microbiome Reflects Diagnosis and Predicts Disease Severity in Paediatric Onset Inflammatory Bowel Disease. Scand. J. Gastroenterol. 54 (8), 969–975. doi: 10.1080/00365521.2019.1644368 31329473

[B19] Manandhar I. Alimadadi A. Aryal S. Munroe P. B. Joe B. Cheng X. (2021). Gut Microbiome-Based Supervised Machine Learning for Clinical Diagnosis of Inflammatory Bowel Diseases. Am. J. Physiol. Gastrointest Liver Physiol. 3, 00360.2020. doi: 10.1152/ajpgi.00360.2020 PMC8828266 33439104

[B20] Mancabelli L. Milani C. Lugli G. A. Turroni F. Cocconi D. van Sinderen D.. (2017). Identification of Universal Gut Microbial Biomarkers of Common Human Intestinal Diseases by Meta-Analysis. FEMS Microbiol. Ecol. 93 (12), fix153. doi: 10.1093/femsec/fix153 29126267

[B21] Members C.-N. Partners. (2021). Database Resources of the National Genomics Data Center, China National Center for Bioinformation in 2021. Nucleic Acids Res. 49 (D1), D18–D28. doi: 10.1093/nar/gkaa1022 33175170 PMC7779035

[B22] Molodecky N. A. Soon I. S. Rabi D. M. Ghali W. A. Ferris M. Chernoff G.. (2012). Increasing Incidence and Prevalence of the Inflammatory Bowel Diseases With Time, Based on Systematic Review. Gastroenterology 142 (1), 46–54.e42; quiz e30. doi: 10.1053/j.gastro.2011.10.001 22001864

[B23] Oliveira S. B. Monteiro I. M. (2017). Diagnosis and Management of Inflammatory Bowel Disease in Children. BMJ 357, j2083. doi: 10.1136/bmj.j2083 28566467 PMC6888256

[B24] Pittayanon R. Lau J. T. Leontiadis G. I. Tse F. Yuan Y. Surette M.. (2020). Differences in Gut Microbiota in Patients With *vs* Without Inflammatory Bowel Diseases: A Systematic Review. Gastroenterology 158 (4), 930–946.e931. doi: 10.1053/j.gastro.2019.11.294 31812509

[B25] Rahman S. F. Olm M. R. Morowitz M. J. Banfield J. F. (2018). Machine Learning Leveraging Genomes From Metagenomes Identifies Influential Antibiotic Resistance Genes in the Infant Gut Microbiome. mSystems 3 (1), e00123–17. doi: 10.1128/mSystems.00123-17 PMC5758725 29359195

[B26] Rajca S. Grondin V. Louis E. Vernier-Massouille G. Grimaud J. C. Bouhnik Y.. (2014). Alterations in the Intestinal Microbiome (Dysbiosis) as a Predictor of Relapse After Infliximab Withdrawal in Crohn's Disease. Inflamm. Bowel Dis. 20 (6), 978–986. doi: 10.1097/mib.0000000000000036 24788220

[B27] Rooks M. G. Garrett W. S. (2016). Gut Microbiota, Metabolites and Host Immunity. Nat. Rev. Immunol. 16 (6), 341–352. doi: 10.1038/nri.2016.42 27231050 PMC5541232

[B28] Salem F. Kindt N. Marchesi J. R. Netter P. Lopez A. Kokten T.. (2019). Gut Microbiome in Chronic Rheumatic and Inflammatory Bowel Diseases: Similarities and Differences. United Eur. Gastroenterol. J. 7 (8), 1008–1032. doi: 10.1177/2050640619867555 PMC6794689 31662859

[B29] Schirmer M. Garner A. Vlamakis H. Xavier R. J. (2019). Microbial Genes and Pathways in Inflammatory Bowel Disease. Nat. Rev. Microbiol. 17 (8), 497–511. doi: 10.1038/s41579-019-0213-6 31249397 PMC6759048

[B30] Shaw K. A. Bertha M. Hofmekler T. Chopra P. Vatanen T. Srivatsa A.. (2016). Dysbiosis, Inflammation, and Response to Treatment: A Longitudinal Study of Pediatric Subjects With Newly Diagnosed Inflammatory Bowel Disease. Genome Med. 8 (1), 75. doi: 10.1186/s13073-016-0331-y 27412252 PMC4944441

[B31] Sokol H. Pigneur B. Watterlot L. Lakhdari O. Bermudez-Humaran L. G. Gratadoux J. J.. (2008). Faecalibacterium Prausnitzii is an Anti-Inflammatory Commensal Bacterium Identified by Gut Microbiota Analysis of Crohn Disease Patients. Proc. Natl. Acad. Sci. U.S.A. 105 (43), 16731–16736. doi: 10.1073/pnas.0804812105 18936492 PMC2575488

[B32] Solis A. G. Klapholz M. Zhao J. Levy M. (2020). The Bidirectional Nature of Microbiome-Epithelial Cell Interactions. Curr. Opin. Microbiol. 56, 45–51. doi: 10.1016/j.mib.2020.06.007 32653776 PMC7744412

[B33] Stanghellini V. Chan F. K. Hasler W. L. Malagelada J. R. Suzuki H. Tack J.. (2016). Gastroduodenal Disorders. Gastroenterology 150 (6), 1380–1392. doi: 10.1053/j.gastro.2016.02.011 27147122

[B34] Su W. Yu Y. Xu X. Wang X. Q. Huang J. B. Xu C. D.. (2021). Valuable Clinical Indicators for Identifying Infantile-Onset Inflammatory Bowel Disease Patients with Monogenic Diseases. World J. Gastroenterol. 27 (1), 92–106. doi: 10.3748/wjg.v27.i1.92 33505153 PMC7789064

[B35] Turner D. Hyams J. Markowitz J. Lerer T. Mack D. R. Evans J.. (2009). Appraisal of the Pediatric Ulcerative Colitis Activity Index (PUCAI). Inflamm. Bowel Dis. 15 (8), 1218–1223. doi: 10.1002/ibd.20867 19161178

[B36] van Rheenen P. F. Aloi M. Assa A. Bronsky J. Escher J. C. Fagerberg U. L.. (2021). The Medical Management of Paediatric Crohn's Disease: An ECCO-ESPGHAN Guideline Update. J. Crohns Colitis. 15, 171–194. doi: 10.1093/ecco-jcc/jjaa161 33026087

[B37] Wang Y. Gao X. Ghozlane A. Hu H. Li X. Xiao Y.. (2018b). Characteristics of Faecal Microbiota in Paediatric Crohn's Disease and Their Dynamic Changes During Infliximab Therapy. J. Crohns Colitis 12 (3), 337–346. doi: 10.1093/ecco-jcc/jjx153 29194468

[B38] Wang Y. Gao X. Zhang X. Xiao F. Hu H. Li X.. (2021). Microbial and Metabolic Features Associated With Outcome of Infliximab Therapy in Pediatric Crohn's Disease. Gut Microbes 13 (1), 1–18. doi: 10.1080/19490976.2020.1865708 PMC7808429 33430702

[B39] Wang X. Q. Xiao Y. Xu X. Yu Y. Shan C. Y. Guo Y.. (2018a). Study of Disease Phenotype and Its Association With Prognosis of Paediatric Inflammatory Bowel Disease in China. BMC Pediatr. 18 (1), 229. doi: 10.1186/s12887-018-1212-x 30001197 PMC6044010

[B40] Wang X. Q. Zhang Y. Xu C. D. Jiang L. R. Huang Y. Du H. M.. (2013). Inflammatory Bowel Disease in Chinese Children: A Multicenter Analysis Over a Decade From Shanghai. Inflamm. Bowel Dis. 19 (2), 423–428. doi: 10.1097/MIB.0b013e318286f9f2 23340680

[B41] Xue A.-J. Miao S.-J. Sun H. Qiu X.-X. Wang S.-N. Wang L.. (2020). Intestinal Dysbiosis in Pediatric Crohn's Disease Patients With IL10RA Mutations. World J. Gastroenterol. 26 (22), 3098–3109. doi: 10.3748/wjg.v26.i22.3098 32587451 PMC7304104

[B42] Xu C. Zhou M. Xie Z. Li M. Zhu X. Zhu H. (2021). LightCUD: A Program for Diagnosing IBD Based on Human Gut Microbiome Data. BioData Min 14 (1), 2. doi: 10.1186/s13040-021-00241-2 33468221 PMC7816363

[B43] Yang W. H. Heithoff D. M. Aziz P. V. Sperandio M. Nizet V. Mahan M. J.. (2017). Recurrent Infection Progressively Disables Host Protection Against Intestinal Inflammation. Science 358 (6370), eaao5610. doi: 10.1126/science.aao5610 29269445 PMC5824721

[B44] Zhou Y. Chen H. He H. Du Y. Hu J. Li Y.. (2016). Increased Enterococcus Faecalis Infection Is Associated With Clinically Active Crohn Disease. Med. (Baltimore) 95 (39), e5019. doi: 10.1097/MD.0000000000005019 PMC5265965 27684872

[B45] Zhou Y. Xu Z. Z. He Y. Yang Y. Liu L. Lin Q.. (2018). Gut Microbiota Offers Universal Biomarkers Across Ethnicity in Inflammatory Bowel Disease Diagnosis and Infliximab Response Prediction. mSystems 3 (1), e00188–17. doi: 10.1128/mSystems.00188-17 29404425 PMC5790872

